# Editorial: Emerging Infectious and Vector-Borne Diseases: A Global Challenge

**DOI:** 10.3389/fpubh.2020.00214

**Published:** 2020-07-07

**Authors:** Katherine M. Warpeha, Vincent Munster, Catherine Mullié, Shu Hui Chen

**Affiliations:** ^1^Department of Biological Sciences, University of Illinois at Chicago, Chicago, IL, United States; ^2^National Institute of Allergy and Infectious Diseases (NIAID) and National Institutes of Health, Bethesda, MD, United States; ^3^Laboratoire AGIR (Agents Infectieux, Résistances et Chimiothérapie), EA UPJV 4294, University of Picardy Jules Verne, Amiens, France; ^4^National Institutes of Health (NIH), Bethesda, MD, United States

**Keywords:** infectious disease, vector-borne, zoonotic, viral, containment, diagnostics, climate change, plant-animal interaction

This Special Research Topic focused on global challenges in identification, transfer, spread, treatment, and containment of infectious and vector-borne diseases (including re-emerging infectious diseases). Research, medical and community leaders have come together to address infectious and zoonotic or vector-borne diseases by making a public and proactive shift from reactionary to pre-emptive approaches to address diseases. Eight articles of current importance were published in this special issue.

The World Health Organization's *Global vector control response 2017–2030 (GVCR)*^1^ has directed a new strategy to strengthen vector monitoring and control worldwide, stating that through increased capacity, improved surveillance and response in coordinating and integrating actions, disease can be prevented/managed. In 2017, the World Health Assembly adopted a resolution (WHA 70.16: An integrated approach for the control of vector-borne diseases^2^) asking Member States for strategies to implement vector control. 2020 goals include Reduction in: (i) mortality due to vector-borne diseases globally relative to 2016 (by 30%), (ii) case incidence due to vector-borne diseases globally relative to 2016 (by 25%), and to ask of all countries to contain spread of disease to prevent epidemics of vector-borne diseases. This issue includes articles highlighting host immune responses and development of biomarkers that may assist in disease management. Wang et al., shows that host antibodies to the Plasmodium TatD-like DNase protected mouse models from infection (Wang et al.). The identification of the serologic biomarker, SjSP-13, show promise as diagnostics and may prove valuable for surveillance and containment (Xu et al.).

Many emerging and re-emerging human infectious diseases have animal origins as described in the Global Infectious Disease and Epidemiology Network (GIDEON)^3^ database. Yet, zoonotic hosts, distribution and incidences of disease are still poorly understood. Difficulty in managing infectious diseases lies in how humans use land and water. Urbanization and deforestation resulting in loss of habitat for existing plants and animals have placed humans and animals in closer contact under unnatural circumstances increasing the human-animal interface. Changing patterns of land use and the increase in international travel, trade, and shipping bring human populations into increased contact with various vectors that travel with humans and transported goods, including completely different biomes, from tropical to temperate. Global and local climatic alterations and other environmental changes have only intensified this matter, with the potential for worldwide spread of certain vector species. The collective geographic ranges of all known host species further complicates the issues of disease, as the range of many vectors is now greatly expanded due to changes in climate and increase of transport connectivity, allowing invasive species to cross borders more readily. In this issue, Sklenovská and Ranst explores the emergence, epidemiology, and ecology of the zoonic disease, monkeypox, from 1970 to 2018 to identify gaps in surveillance and containment and spread of the disease outside of traditional geographic areas. This is only one example of the many contributing factors to the spread of infectious diseases. The development of predictive models for future zoonotic/vector-borne disease incidence by using established cross-disciplinary tools integrating both classic and novel tools (e.g., epidemiology and machine learning) should be at the forefront of research.

Types of disease models require specific study and attention for worldwide reduction. Viral diseases continue to be a major threat to health and safety in the twenty-first century for both human and wild or domestic animal species. A more comprehensive One Health framework, using environmental data such as land, water, anthropogenic data (population growth, spatial connectivity) and vector and host distribution is required. This is underscored by the recent emergence and re-emergence of Ebola virus, avian influenza H5N1 & H7N9, Nipah and Hendra viruses, severe acute respiratory syndrome coronavirus (SARS-CoV), Middle East respiratory syndrome (MERS) CoV, Zika, the current coronavirus outbreak, and the continuous increased controllable viral outbreaks due to lack of immunization (e.g., measles). Locality mapping of Zika virus (ZIKV) shows the migration of the virus from Africa to Asia and further suggests changes that occurred in different lineages result in vastly different disease outcomes: one associated with microcephaly and one that is not (Khaiboullina et al.). This study exemplifies how little is known about interactions of viruses with hosts. In addition, changes in the virus-host ecology that results in cross-species contact and transmission will require much time and financial investment for adequate study in the field, the lab, and in populations.

Topics touch upon other vastly different problems facing public health such as disease dissemination, treatment, emergence, and re-emergence of diseases, historical surveys to understand disease migration, etc. as it pertains to globalization and climate change. Malaria Vector *A. stephensi* and the anthropogenic factors driving range expansion suggests a re-emerging threat in certain regions (Surendran et al.). Understanding disease progression is imperative to treatment of multi-drug resistant diseases. Akinsola et al. identified factors related to sputum conversion which can aid clinical prediction and outcomes for tuberculosis. Genetics has proven to be a powerful tool for understanding spread, etiology of disease, and search for better treatments and diagnostics. Zhang et al. explores genetic diversity of *C. sinensis* from human fecal samples and found that internal transcribed spacer regions in rDNA has a low degree of genetic diversity and may provide a valuable tool for epidemiological studies. Li et al. tested the efficacy of two HA proteins as a possible bivalent vaccine in chickens for H5 and H7N9 pathogenic avian influenza viruses.

This Research Topic Emerging Infectious and Vector-Borne Diseases: A Global Challenge calls for more pre-emptive approaches to mitigating infectious diseases as climate change (deforestation/invasive plants, urbanization and globalization) blurs boarders for disease causing agents (e.g., viral spread through increased modes of human transportations or climate changes that allow for vectors including plant hosts to migrate to new regions). Future risk requires baseline data worldwide. Information should be collected routinely regarding geographic, taxonomic (with respect to animal reservoirs) occurrence of vectors, and for human populations to assess disease risks and spread. Lastly, the environment itself requires study in order to assess best practices to prevent “edge effect,” the transfer of viruses, bacteria, fungi, and other parasites. We hope that this issue raises thought provoking ideas and science moving forward.

## Author Contributions

All authors listed have made a substantial, direct and intellectual contribution to the work, and approved it for publication. KW, VM, CM, and SC contributed as monitoring editors for this special topics issue. SC contribution was in affiliation with her previous appointment at the U.S. National Institute of Allergy and Infectious Diseases.

## Conflict of Interest

The authors declare that the research was conducted in the absence of any commercial or financial relationships that could be construed as a potential conflict of interest.

## Footnotes

^1^https://www.who.int/vector-control/publications/global-control-response/en/

^2^https://www.who.int/neglected_diseases/mediacentre/WHA70_R16_Eng.pdf?ua=1

^3^https://www.gideononline.com/

